# Nine out of ten trauma calls to a Norwegian hospital are avoidable: a retrospective analysis

**DOI:** 10.1186/s12873-015-0026-5

**Published:** 2015-02-03

**Authors:** Harald Stordahl, Eva Passas, Andreas Hopland, Erik Waage Nielsen

**Affiliations:** Department of Anaesthesia and Intensive Care, Nordland Hospital Bodø, Bodø, Norway; The Universities of Nordland and Tromsø, Tromsø, Norway; The University of Tromsø, Tromsø, Norway; Department of Surgery, Nordland Hospital Bodø, Bodø, Norway; Department of Prehospital Medical Services, Nordland Hospital Bodø, Bodø, Norway

**Keywords:** Trauma team activation, Overtriage, Time lapse, Healthcare resource utilization

## Abstract

**Background:**

Our aim was to estimate the degree of overtriage (Injury Severity Score [ISS] ≤ 15) of trauma call patients in Nordland Hospital Bodø, Norway. We also determined the transportation time from injury to hospital admission.

**Methods:**

We used data from our Acute Medical Information System, ambulance records and patient charts relating to ISS and estimation of response and transport times. Data were collected for all trauma call patients in the period from the establishment of the trauma call system in June 2008 until the 31st of December 2010.

**Results:**

We identified 421 out of 458 possible trauma call patients with sufficient clinical information available for ISS scoring. Of these 385 had an ISS ≤15. Overtriage was 91.5% (95% CI: 88.8%–94.2%). Median time from injury to the arrival of transport, and from injury to arrival in hospital, was 36 minutes and 1 hour 27 minutes, respectively.

**Conclusions:**

To our knowledge 91.5% is the highest overtriage ever published. There is a need for narrowing the trauma call criteria. This could be achieved by implementing clinical observations during the long transportation time.

## Background

When a trauma call is activated in our hospital (Nordland Hospital Bodø, Norway), a full trauma team is scrambled. The criteria for activation are shown in Table 1. The trauma team consists of physicians, nurses and allied health personnel. The implementation of a trauma call system is an important and life saving measure in the treatment of severely injured patients [1], but is resource intensive, placing a great strain on on-call resources, and increasing waiting times for other patients. It is therefore essential to use the trauma team where it is really needed. We wished to determine how often our trauma call patients had minor injuries, defined as those with an Injury Severity Scores (ISS) of 15 or lower. The Committee on Trauma, American College of Surgeons use an Injury Severity Score of 16 or more to designate correctly transported patients to a trauma centre, and this limit is widely used in publications on trauma treatment [1].

**Table 1:** **Trauma team activation criteria at Nordland Hospital Bodø**

**Criteria category**	**Criterion**
Mechanism of injury	Co passenger dead
	Trapping in wreck
	Wreck deformity
	Ejected from vehicle
	Pedestrian thrown upon car, or through air
	Children hit by car with speed exceeding 30 km/h
	Fall from > 5 m
	Motorcycle accident
Extent of injury	Shot or stab wounds
	Large bleeding
	Large crush injury
	Suspected pelvic injury
	Two large fractures
	Burn injury > 15% body surface
	Burn injury with inhalational injury
Vital parameters	Disturbed respiration
	Tachycardia > 120
	Loss of consciousness
	Hypotension

Northern Norway is sparsely populated and the distances are vast. We therefore also evaluated the time elapsed from when the accident occurred to when the patient arrived at the hospital in order to determine whether we can make better use of clinical observations during long transports, in order to minimise overtriage.

## Methods

This study was performed at Nordland Hospital, Bodø, a regional hospital providing secondary care. Nordland Hospital is one of eleven acute care hospitals in Northern Norway. The hospital admits trauma patients, predominantly from road traffic accidents (data not shown), via thirty ambulances, five sea ambulances and a SeaKing rescue helicopter with a fully trained anaesthetist based in Bodø.

The majority of trauma patients are treated in Nordland Hospital Bodø. Neurosurgical injuries and severe thoracic injuries, however, are sent via air ambulance to a tertiary-level of care hospital in Tromsø.

We acquired information from the acute medical information system AMIS (Akuttmedisinsk Informasjonssystem, Nirvaco, Norway), ambulance charts and the digital in-hospital patient chart system DIPS (Distribuert Informasjons og Pasientdatasystem, DIPS ASA, Norway). From AMIS we extracted data on all 458 trauma call patients in the period from when the trauma call system was started in June 2008 until 31st December 2011. The data were anonymised and transferred to an Excel file for autofilter search and pivot tables. Two doctors experienced in treating trauma, one anaesthetist and one surgeon independently scored the patients using the ISS and the Abbreviated Injury Score (AIS).

The AIS classifies the patient’s injury in a numerical scale from 1 to 6 where 1 represents the mildest form of injury and 6 represents a lethal injury. The ISS is calculated by summing the squares of the three highest AIS scores in different body regions. The two doctors used the AIS ©2005, Updated 2008 as a guideline for scoring the patients. Like Uleberg et al. we defined overtriage as 1 minus the positive predictive value, where the positive predictive value was the probability of serious injury conditional on trauma team activation [2].

The time intervals between the report of an accident and the start of transport, and between the report of an accident and arrival at the hospital were obtained from ambulance charts and the Emergency Medicine Communication Central (EMCC).

Ethical approval was given by the Regional Committees for Medical and Health Research Ethics, with project number 2012/1889.

## Results

**Figure 1:**
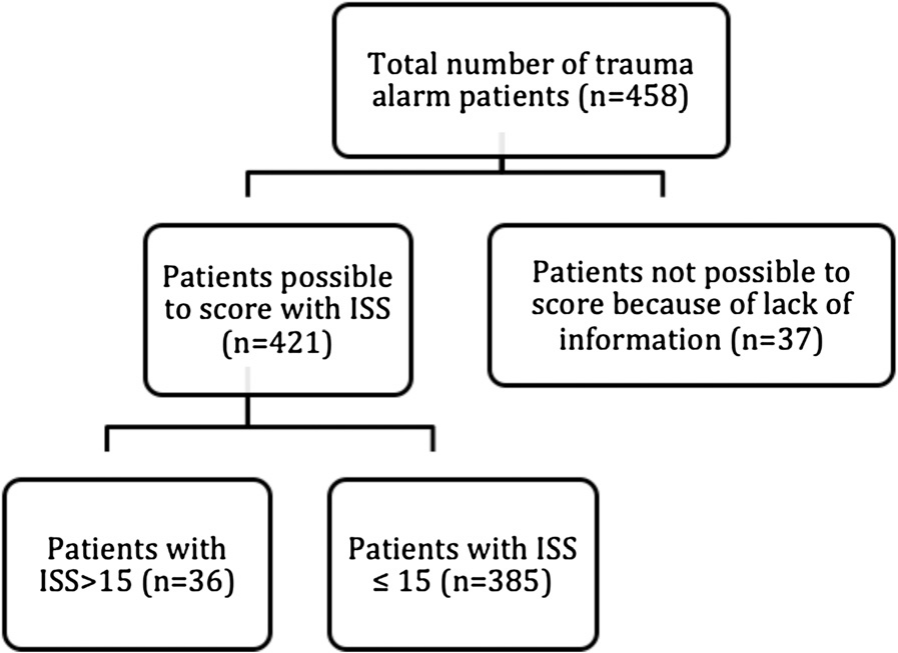
**Flow chart of patient inclusion criteria and ISS scoring.**

**Figure 2:**
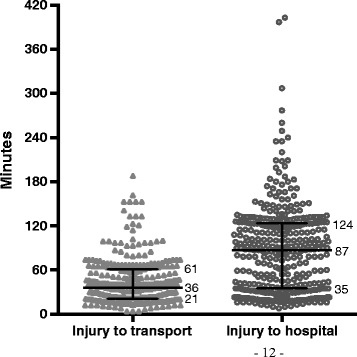
**Prehospital time use.** Minutes from time of injury to start of transport and from injury to hospital admittance. Horizontal bars are median with 25–75th percentile range.

We identified 421 trauma call patients with sufficient clinical information available for ISS scoring out of a possible 458 (Figure 1). Of these patients 385 had an ISS ≤15 on admission, which means the overtriage was 91.5%. Median time from injury to transportation and from injury to admission with a 25–75th percentile range are presented in Figure 2.

## Discussion

We found a very high proportion of unnecessary trauma calls in our hospital. Although the numerous smaller hospitals receive most trauma call patients in Norway, these hospitals publish few studies on trauma call triage. If our findings from a smaller hospital are representative, a substantial national overtriage exists. Studies from larger tertiary level of care hospitals have also shown that a large proportion of patients are overtriaged [2,3]. A trauma call usually occupies a large number of on-call staff. In smaller hospitals, almost all on-call staff will be involved in a trauma call. In our hospital this accounts for a minimum of 11. In the meantime other patients, including other emergencies, will have their diagnostic procedures, radiology investigations, blood samples or operations postponed. If the emergency room is full during a trauma call, many patients will be sent to a medical or surgical ward before they have been fully examined and had their treatment plan developed in the emergency room. This is done to provide enough space for the trauma team. The overuse of trauma calls is often considered a good training situation for the trauma team. Medical training on patients, however, should only be performed after informed consent has been obtained. It is also a legitimate concern that continuous overtriage will negatively affect the trauma team’s responsiveness and motivation.

The majority of trauma call patients undergo a full body CT scan that will give them a radiation dose of more than 20 millisievert. This is twice the level required to give a 40-year-old adult a 1/1000 chance of future cancer, as defined by the National Academy of Science’s Seventh Assembly of the Committee on Biologic Effects of Ionizing Radiation [4]. The radiation dose alone is therefore a valid reason to limit the amount of trauma call patients with low ISS scores routinely undergoing CT scans. The radiologist and countersigning radiologist are also given a substantial extra workload examining the CT scans.

We also found that, in the majority of cases, no trauma call criteria are registered. If a criterion is registered, it is usually “mechanism of injury” (MOI). It is well known that MOI as trauma team activation criteria will give a high overtriage rate [2,3,5]. By using only MOI criteria Uleberg et al. found the same overtriage rate in Trondheim as in our study [2]. MOI was employed by 38 hospitals (83%) in Norway in 2008 as a reason for activation of the full trauma team [6]. We believe new criteria for full trauma team activation must be based on vital parameters and clinical findings. This might be possible without an unacceptable increase in undertriage [2,3,5]. It is also important that the EMCC hits the call based on these criteria, without conference with in-hospital doctors on-call.

Even today there is no consensus as to which criteria should be used for trauma team activation [1]. It is therefore possible that our ISS limit would prevent some patients with lesser injuries from the benefit of a trauma team. However, the perception that overtriage burdens the hospital adversely affects the treatment of other patients and is a poor use of resources is spreading [1].

It is difficult to define an appropriate level of overtriage. Jenkins et al. [7] suggests that to achieve an undertriage rate of 5–10%, an overtriage rate of 30–50% may be needed. DiDomenico et al. [8] refers to The American College of Surgeons Committee of Trauma that suggested that 50% overtriage is necessary to maintain an acceptable undertriage rate, but also implies that there are no benchmark rates of overtriage and undertriage.

We found a median prehospital time of 1 hour and 27 minutes compared with 44 minutes, including both ambulance and helicopter, reported by the tertiary level of care Ullevål University Hospital in Oslo (NO Skaga, personal communication, Feb 2013). The trauma call system in Norway was first introduced at tertiary level of care university hospitals when there were shorter transport distances and times. Today, however, the majority of trauma call patients are admitted to hospitals with longer prehospital time use. The trauma centre at St. Olav’s University Hospital in Trondheim reported in 2009 a median transport time of 1 hour and 16 minutes from when the accident occurred until arrival at the hospital. This is only 11 minutes shorter than our transport time. Therefore, it is reasonable to believe that the great majority of trauma call considerations in Norway have time to make use of clinical observations during long transports in order to minimise overtriage. This possibility has not been studied in Norway so far, even though several authors have concluded that a patient without any clinical symptoms involved in a high-energy accident does not necessitate the use of trauma team activation [2].

## Conclusions

To our knowledge, an overtriage rate of 91.5% is the highest reported to date. The trauma call criteria must focus more on clinical findings and less on mechanism of injury. Clinical observations from the long transport time could be used to reduce overtriage.
